# Case Study Protocol to Evaluate the Impact of Training Intervention on Cleaners’ Knowledge Level, Perceptions and Practices regarding Correct Cleaning Techniques at Selected Care Facilities in Limpopo Province, South Africa

**DOI:** 10.3390/nursrep14010025

**Published:** 2024-01-31

**Authors:** Takalani Grace Tshitangano

**Affiliations:** Department of Public Health, Faculty of Health Sciences, University of Venda, Thohoyandou 0950, South Africa; takalani.tshitangano@univen.ac.za; Tel.: +27-824484111

**Keywords:** cleaning, health facilities, impact, knowledge, practices, staff, training intervention

## Abstract

Despite being preventable, healthcare-associated infections are known primary causes of patient mortality and morbidity, threatening global public health. Though it is believed that one competent and dedicated cleaning staff member given the right tools and enough time can prevent more health-care-associated infectious diseases than a room full of doctors and nurses can cure, it was discovered in Letaba Hospital of the Limpopo Province, South Africa, that knowledge and practices of infection control among cleaning staff were not optimal. The proposed study aims to evaluate the impact of training interventions on cleaners’ knowledge levels and practices. In Phase 1, cleaners’ pre-training knowledge level, practices, and perceptions regarding correct cleaning techniques will be assessed through qualitative, individual, in-depth interviews. The initial question will read, “Describe the steps you follow when cleaning at this health facility and why?” In Phase 2a, a cleaning training program will be developed based on the South African Qualification Authority ID 118730 Healthcare Cleaner: Occupational Certificate Curriculum and the Center for Diseases Control and ICAN’s Best Practices for Environmental Cleaning in Healthcare Facilities in Resource-Limited Settings. In Phase 2b, a cleaning training intervention will be implemented. In Phase 3, cleaners’ post-training knowledge level, practices, and perceptions will be reassessed and compared to pre-training findings. The Standard Protocol Items: Recommendations for an Interventional Trial, commonly known as SPIRIT, guided the development of this protocol. This protocol received ethical clearance number FHS/22/PH/04/3005 in August 2023 from the University of Venda Human and Clinical Trials Research Ethics Committee. The protocol approval was granted by the Limpopo Provincial Department of Health (LP_2022-05-028) in October 2023. This protocol is registered with the South African National Clinical Trial Registry. The findings of this study may provide baseline data upon which healthcare facilities’ cleaner training qualification curriculum may be developed. In addition, this protocol contributes to the application of qualitative methodology in an intervention trial.

## 1. Introduction 

Healthcare-associated infections (HCAIs) remains the most frequently occurring adverse consequence of healthcare, a principal cause of diseases, and the second leading cause of death worldwide [1]. Scientific reports indicate that for every one hundred (100) patients admitted to care facilities, seven in high-income countries and ten in low-income countries acquire at least one type of HCAI. Healthcare-associated infections (HAIs), or nosocomial infections, are infections acquired while receiving medical or surgical treatment in a care facility [1]. According to the World Health Organization (WHO), HAIs are infections occurring in patients during the process of care in a facility which were not present or incubating during admission. These include infections appearing after discharge and occupational infections among the staff of the facility [2]. In South Africa, the overall occurrences of HAIs at one hospital over a period of one month in 2018 was 7.67% [3] compared to Ethiopia (16.940), China (3.12%), Morocco (10.3%), and Botswana (13.54%) [4]. Lloyd, Bekker, Van Weissenbruch, et al. [5] found that HAIs are main causes of neonatal diseases and deaths in South Africa. An estimated 6.5% of patients in acute care hospitals had at least one HAI in the European Union and European Economic Area (EU/EEA) [6]. In Australia, an estimated 170,574 HAIs occurred among adults admitted to public hospitals in 2018, resulting in 7583 deaths, compared to a 6.8% overall prevalence among inpatients at six hospitals in Saudi Arabia on 11 May 2017 (Lydeamore, Mitchell, Bucknall, et al. [7]). 

The Center for Diseases Control [8] highlights that the transmission of HAIs is often influenced by environmental contamination in healthcare settings. According to the CDC [8], a room that has been previously occupied by an infected or colonized patient increases the risk of the next patient’s colonization and infection. These claims emphasize the importance of the immediate care of patients’ environments, particularly surfaces within the patient’s proximity, frequently touched by or in direct physical contact with the patient (e.g., bed rails, bedside tables, and chairs, etc.) in preventing the subsequent transfer of microorganisms [9]. What makes taking care of patients’ immediate environmental surfaces critical is that some microbes can survive on surfaces for months, with the actual survival times varying based on such factors as humidity, temperature, and surface type [10]. Acinetobacter spp., for example, can survive up to 5 months, while Klebsiella spp. can last up to 30 months. As recommended by many previous studies [4,7,11,12], environmental surface cleaning is a critical intervention for HAI prevention and control in healthcare facilities.

According to Gon, Kabanywanyi, Blinkhof, et al. [13], environmental surface cleaning is the systematic removal of microbes from surfaces. This process reduces direct bacterial and viral transmission to patients and indirect transmission via medical equipment or the hands of healthcare workers. Hicks [14] argues that one competent and dedicated cleaning staff member, if given the right tools and enough time, may prevent more infectious diseases than a room full of doctors and nurses can cure. However, Gon, Kabanywanyi, Blinkhof, et al. [12] reported that environmental hygiene, particularly surface cleaning, remains poor in many SA hospitals despite the availability of the National Infection Prevention and Control (IPC) Strategic Framework [15], which outlines the standards for infection and control in healthcare facilities; the NDOH Practice Manual for the implementation of the IPC strategic framework [16], which outlines step-by-step guidance on how IPC programs should be run in healthcare facilities; and the CDC [8], which outlines cleaning techniques per hospital ward and procedure. Inadequate education of staff and inadequate in-service IPC training and supervision were identified as barriers to IPC in care facilities in resource-limited settings [15]. Cleaning staff’s knowledge and implementation of the two above-mentioned IPC frameworks becomes imperative, as correct cleaning procedures can go a long way towards preventing the occurrence of HAIs in healthcare facilities. 

Although there is a dearth of published evidence on the status of poor environmental hygiene, the barriers faced by cleaners within LMICs are indicative of wider neglect. There are limited data pertaining to the knowledge level of cleaning staff in South Africa. Thus, Peta’s [17] is the only knowledge study conducted among cleaning staff in Limpopo province, which discovered in Letaba Hospital of South Africa that knowledge and practices of infection control among cleaning staff were not optimal in 2014. Thorough cleaning is important for infection control, whereas inadequate knowledge and incorrect cleaning methods carry increased risks of HAI transmission in healthcare settings. Thus, cleaning staff knowledge and practices need to be constantly assessed. This paper is dedicated to assessing cleaning staff knowledge level, perceptions, and practices regarding correct cleaning methods before and after training interventions in selected healthcare facilities of Limpopo province. The findings of this study will serve as a baseline upon which cleaning training programs may be developed or reviewed in the study setting and beyond. 

## 2. Literature Review

Environmental surface cleaning is globally regarded as a foundational intervention for the prevention and control (IPC) of infection, including healthcare-associated infections (HAIs) [8]. Environmental surface cleaning is a multifaceted intervention involving surface cleaning and disinfection [8]. According to Better Care [6], environmental surface cleaning refers to the removal of visible dirt, dust, and debris in patients’ surroundings in care facilities including clinical and administrative areas. This is based on the belief that dust in patients’ surroundings contains shed skin cells and microbes, which can be spread on surfaces and in the air when sweeping or dry dusting. In addition, items like door handles, buttons/knobs on medical equipment, light switches, and patient monitors are frequently touched by healthcare workers and patients. Thus, the ability of these surfaces to hold microbes that are transferred from people’s hands pose a high risk for cross-transmission. Therefore, Gon, Kabanywanyi, Blinkhof, et al. [13] emphasize that a most important yet neglected aspect of infection prevention is environmental hygiene, particularly surface cleaning. According to the CDC [8], surface cleaning alone results in maximal reductions in environmental contamination, which is the primary cause of patient morbidity and mortality and is currently threatening global public health. 

HAIs often receive public attention only during epidemics. Thus, no institution or country can claim to have eliminated HAI transmission, despite considerable efforts [18]. According to the WHO [2], the prevalence of healthcare-associated infections in developed countries varies between 3.5% and 12%. The European Center for Disease Prevention and Control (EUCDC) reports an average prevalence of 7.1% in Europe. It is estimated that 4,131,000 patients are affected by approximately 4,544,100 episodes of HAIs every year in Europe [8]. Dramowski, Cotton, and Whitelaw [18] concur with the CDC reports and indicate that HAIs are frequent and serious complications affecting 4–8% of admitted neonates and children in high-income countries. 

On the contrary, analysis by the WHO [2] found that HAIs are more frequent in resource-limited settings compared to developed countries. Thus, at one time, the prevalence of HAIs varies between 5.7% and 19.1% in low- and middle-income economies (LMIC). The proportion of patients with ICU-acquired infections ranges from 4.4% to 88.9%, which is equivalent to overall infections rate of 42.7 episodes per 1000 patient days, almost three times higher than in high-income economies [19]. According to Reddy, Bekker, Whitelaw, Esterhuizen, and Dramowski [20], the overall burden of neonatal infection is 3–20 times higher than in high-income country settings. The rates of nosocomial infections in LMIC neonatal intensive care units (ICUs) range from 15 to 62 infections per 1000 patient-days. Though the burden of HAIs is not known in South Africa (SA), it is documented that bloodstream infection (BSI) causes significant diseases and deaths among children. Essel, Tshabalala, Ntshoe, Mphaphuli, et al. [19] reported a total incidence risk of healthcare-associated BSIs of 6.8 cases per 100 admissions for the period between January 2017 and March 2018, and 10.1 cases per 100 admissions for the period between April and September 2018 in Gauteng province of SA. Govender, Todd, Nel, et al. [21], claim that candida is the primary cause of healthcare-associated BSIs in SA, with an estimated national incidence risk of 84 cases per 100,000 hospital admissions from 2016 to 2017 (95% CI 81–86). Interestingly, Nogbou, Phofa, Nchabeleng, Musyoki, et al. [22,23] identified multidrug-resistant (MDR) Acinetobacter baumannii as a leading cause of HAIs in SA with high mortality rates. 

Asante, Hetsa, Amoako, Abia, Bester, et al. [24] report that coagulase-negative staphylococci (CoNS) are increasingly attributed to HAIs, specifically in immunocompromised individuals and those with invasive medical devices, posing a significant concern in SA. What is worrying is that Escherichia coli and Klebsiella spp. isolates from nine healthcare facilities in SA were confirmed in 2016 and 2017 to be colistin-resistant [25]. What is more concerning is that HAIs are a major issue for patient safety and healthcare costs [2]. According to the WHO [2], annual direct costs in Europe due to healthcare-associated infections is estimated at approximately EUR 7 billion, including 16 million extra days of hospital stay, compared to USD 6.5 billion in the USA; whereas similar costs are poorly and differently reported in low- and middle-income economies, including SA. 

According to the WHO [2], several factors can cause HAIs, e.g., prolonged and inappropriate use of invasive devices and antibiotics; insufficient application of standard, high-risk and sophisticated procedures; immuno-suppression and other severe underlying patient conditions; and isolation precautions. Different factors are associated with HAIs in resource-limited settings, namely inadequate environmental hygienic conditions and waste disposal; insufficient equipment; poor infrastructure; understaffing; poor knowledge and application of basic infection control measures; overcrowding; lack of procedures; absence of local and national guidelines and policies; lack of knowledge of injection and blood transfusion safety; lack of surveillance and reporting systems, etc. [26]. This study only focuses on environmental cleaning practices and the knowledge and perceptions of cleaning staff in healthcare facilities.

### Definition of Concepts

Environmental cleaning in healthcare facilities refers to cleaning of general in-patient areas, patient area toilets, patient area floors, spills of blood and bodily fluids, medication preparation areas, sterile services areas, intensive care units (ICUs), special isolation units, burn units, general procedure areas, labor and delivery wards/units, hemodialysis area, pediatric outpatient areas, emergency department, isolation wards, and non-critical patient care equipment, as well as waste management and management of linen and laundry.

Knowledge of cleaning staff refers to correct facts, information, and skills pertaining to correct cleaning procedures of general in-patient areas, patient area toilets, patient area floors, spills of blood and bodily fluids, medication preparation areas, sterile services areas, intensive care units (ICUs), special isolation units, burn units, general procedure areas, labor and delivery wards/units, hemodialysis areas, pediatric outpatient area, emergency departments, isolation wards, and non-critical patient care equipment, as well as waste management and management of linen and laundry (as stipulated in CDC [8], NICD [14], NDOH [15]).

Practices of cleaning staff refer to actual methods used to clean general in-patient areas, patient area toilets, patient area floors, spills of blood and bodily fluids, medication preparation areas, sterile services areas, intensive care units (ICUs), special isolation units, burn units, general procedure areas, labor and delivery wards/units, hemodialysis areas, pediatric outpatient area, emergency departments, isolation wards, and non-critical patientcare equipment, as well as waste management and management of linen and laundry. 

Perceptions of cleaning staff refer to the ways in which cleaning staff regard the correct cleaning procedures of general in-patient areas, patient area toilets, patient area floors, spills of blood and bodily fluids, medication preparation areas, sterile services areas, intensive care units (ICUs), special isolation units, burn units, general procedure areas, labor and delivery wards/units, hemodialysis areas, pediatric outpatient areas, emergency departments, isolation wards, and non-critical patientcare equipment, as well as waste management and management of linen and laundry (as stipulated in CDC [8], NICD [14], NDOH [15]).

## 3. Methodology 

### 3.1. Study Approach and Design 

The development of this protocol was guided by the Standard Protocol Items: Recommendations for Interventional Trials, commonly known as SPIRIT [27]. The study itself will adopt a pre-experimental design using a single group for pre-tests and post-tests through a qualitative methodology, as advocated by Robinson and Mendelson [28] and Steils [29]. The choice of qualitative methodology is informed by the fact that there are very few cleaners available per healthcare facility, ranging from zero to three per clinic, which leads to few participants and a quest to uncover deeply what cleaners in healthcare facilities know and do and why. A cleaning training program will be developed based on the South African Qualification Authority (SAQA) Occupational Certificate: Health Care Cleaner ID 118730 [30] full curriculum (knowledge, practical, and work experience modules), as well as the IPC frameworks of South Africa, which will be used as the intervention and the comparator in this study. One pre-test, in the form of in-depth interviews developed based on the curriculum of this qualification, will be administered by the principal investigator and the research assistant to a single group of cleaners before the training intervention is started, whereas the other post-test will be administered after training to this very group to assess the cleaning knowledge and perceptions of cleaners and compare findings with the pre-test results. Thus, the former test will be given at the start of the intervention, while the latter is given weeks after the intervention. The intervention will be administered for 12 months to cover 120 credits. These twelve-month training periods will be divided into knowledge (39 credits) and practical demonstrations (35 credits) to be delivered in class, while the remaining 46 credits allocated to workplace experience will be acquired at cleaners’ places of work as follows:

Knowledge Modules

- Introductory Studies for Healthcare Cleaners;
- Communication in a Healthcare Environment;
- Healthcare Cleaning;
- Personal Hygiene and Safety;
- Healthcare Laundry Handling;
- Healthcare Waste Handling;
- Healthcare Cleaning: Associated Biology and Infection Control.

Practical Skill Modules

- Maintain Personal Hygiene and Infection Control Precautions;
- Provide Cleaning and Sanitation Services in Public Areas and Offices;
- Provide Cleaning and Sanitation Services in Patient Consulting, Treatment Areas, and Wards.

Work Experience Modules

- Conduct Cleaning and Sanitation Services in Public Areas and Offices;
- Conduct Cleaning and Sanitation Services in Patient Consulting, Treatment Areas, and Wards;
- Attending the Handling of Healthcare Laundry Items.

The training is earmarked to start on 3 April 2024, and end on 30 March 2025. However, the training will only be discontinued when all the curriculum content is delivered, successfully assessed, and the prescribed credits are acquired. As indicated under objectives, in Phase 1, the study will describe cleaners’ pre-training knowledge level and perceptions regarding cleaning techniques to prevent and control HAIs in selected healthcare facilities in Limpopo province. In this phase, in-depth individual interviews guided by the initial question will be conducted. The individual interview is particularly useful in this situation as there are key issues to be investigated, but there may be few respondents to them. Findings from this phase will help the researcher identify the knowledge, perceptions, and practice gaps that need to be closed through training. In Phase 2a, a cleaning training program to close the gaps identified in Phase 1 will be developed based on SAQA [20] as well as the IPC frameworks of SA. In Phase 2b, the approved cleaning training intervention will be implemented. In Phase 3, cleaners’ post-training knowledge level, perceptions, and practices regarding correct cleaning techniques to prevent and control HAIs in healthcare facilities in Limpopo province will be reassessed and compared to the pre-training findings. 

### 3.2. Study Setting 

**Figure 1:**
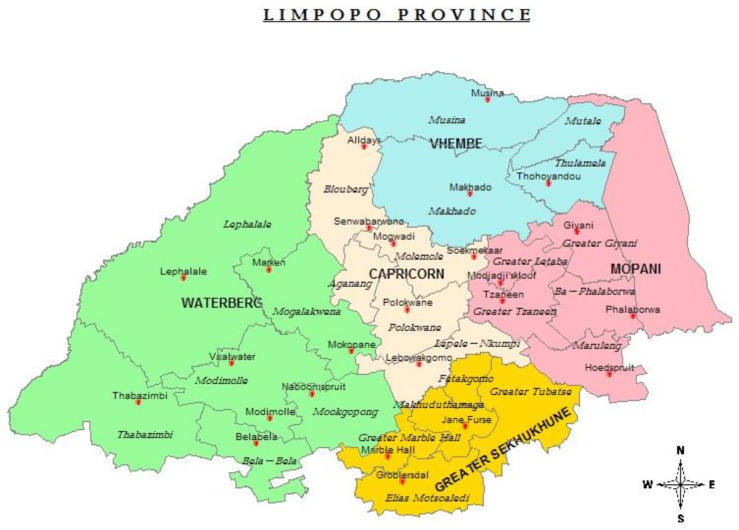
Map of Limpopo Province Health Districts.

This study will take place at selected healthcare facilities in Limpopo Province, South Africa. The chosen facilities are distributed across five districts of this province, as shown in Table 1 and Figure 1.

The map in Figure 1 below shows the health districts of Limpopo province, South Africa.

### 3.3. Outcomes 

Anticipated primary outcomes of this intervention trial will include improved basic cleaning knowledge level, perceptions, and cleaning practices of cleaners, whereas secondary outcomes will include a hygienically clean environment conducive to the reduction in HAIs and their accompanying morbidity and mortality. Cleaning techniques described and practiced by participants will be assigned scores. Thus, correct cleaning procedures will be prepared based on the IPC frameworks of SA to guide the investigator and research assistant in the allocation of assessment scores, where scores of 0–59% will represent an inadequate knowledge level, negative perception, and risky cleaning practices; scores of 60–74% will represent a satisfactory knowledge level, perceptions, and cleaning practices; and scores of 75% and above will represent an adequate knowledge level, positive perceptions, and safe cleaning practices. Improved basic cleaning knowledge level, perceptions, and cleaning practices will be measured in terms of the differences between pre-training scores and post-training scores per participant. Sixty percent and above will be regarded as adequate knowledge, practices, and perceptions, whereas fifty-nine percent and below will be regarded as inadequate knowledge. 

### 3.4. Participant Timeline 

The following sequence of events in Table 2 will take place as scheduled for the intervention trial process.

### 3.5. Population, Sampling, and Eligibility Criteria 

This study is targeting all cleaners with matriculation certificates or cleaning experience coupled with Adult Basic Education Training (ABET) level 4 employed at selected health facilities in Limpopo Province. This targeted population was chosen because the training intervention will be based on SAQA ID unit standard [20], which is in English. Based on the advice of the Provincial Infection Control Directorate and funding constraints, one regional hospital, one district hospital, one specialized hospital, one PHC facility, and one tertiary hospital were randomly selected to ensure that all the districts of the province are represented. Since there are only three specialized hospitals in Limpopo province, the names of all three were placed in a bowl, and a neutral person was made to toss and select one while blindfolded. The same process was followed to select one district hospital, one PHC facility, one tertiary hospital, etc., until all five required facilities were chosen. Thus, the selected facilities include Letaba Regional Hospital from the Mopani District, Jane Furse District Hospital from the Sekhukhune District, Hayani Specialized Hospital from the Vhembe District, Pienaarsrivier PHC Clinic from the Waterberg District, and Mankweng/Turfloop Tertiary Hospital from Capricorn District. Since the number of cleaners per selected facility is not known up front, the total convenience sample size will be made up of all cleaners (male and female, irrespective of confounding factors; from 19 to 64 years old) who are employed by the Limpopo Provincial Department of Health and placed at the selected facilities, and who are available at the time of data collection and who consent to participate in this study, with an estimated number of thirty (30) participants. This will be carried out to avoid selection bias. The importance of this trial to cleaners will be emphasized to motivate cleaners’ enrolment. Thus, all cleaners who will not consent in writing to participate will be excluded. To improve adherence to the intervention protocol, the University of Venda Human and Clinical Trial Research Ethics Committee (HCTREC) monitors the trial implementation processes and requires the principal investigator to apply using a prescribed form for any change to the protocol at any time when a need arises. 

### 3.6. Data Collection Tool 

The independent variable in this study is cleaning training intervention, whereas the dependent variables include cleaners’ knowledge level, perceptions, and cleaning practices. Thus, to describe the cleaning knowledge level, perceptions, and practices of cleaning staff, an in-depth individual interview guide was developed based on the SAQA ID 118730 qualification curriculum. The interview guide is divided into two sections: Section A covers the demographic characteristics of participants; Section B is composed of the main question, which reads “Describe the steps you follow when cleaning at this health facility and why?” Probing questions and correct cleaning procedures will be prepared based on the IPC frameworks of SA to guide the investigator and assistant researcher in the allocation of assessment scores. There will be a minimum of five days allocated for interviews per selected health facility, with the exact number of days dependent on the number of participants per facility. Each interview session will last for about 60 min per participant. All interviews will take place at the selected health facility. The cleaning procedures are also developed based on the SAQA occupational certificate: Healthcare Cleaner ID 118730 [30]. Data will be collected by the principal investigator, who is a professor with more than ten (10) years of research experience, together with a research assistant, who is a Ph.D. candidate. The research assistant will be trained thoroughly in how to take field notes during the interviews. The investigators will keep reminding participants about the importance of the study to cleaners and others to promote retention and complete follow-up. However, for those who discontinue or deviate from the protocol, their outcomes will be assessed up until their exit point. 

### 3.7. Pre-Testing of the Tools 

Pre-testing of data collection tools will take place at Thohoyandou health center among all cleaners who will consent to participate in the study (1) to test whether the content of the instrument is relevant and adequate; (2) to test whether the instrument would elicit responses required to achieve the research objectives; (3) to develop an appropriate procedure for administering the instrument concerning field conditions; and (4) to test whether the wording of questions is clear and suited to the understanding of the participants [31]. During the pre-test, the following will be noted: time to be taken during interviews; the ease of recording or taking notes; conversational flow possibilities; and ambiguities [32]. Any challenges noticed on the tool during the pre-test will be corrected. 

### 3.8. Data Collection and Monitoring Methods 

In Phase 1, the researcher and assistant will recruit participants, obtain informed consent, and conduct interviews using the attached tool only at selected healthcare facilities, as indicated above. 

#### 3.8.1. Data Collection Date, Time, Duration of the Meeting and Venue

The researcher and assistant will schedule the date and time of the meeting and communicate these to the selected healthcare facilities’ managers, requesting their help regarding the venue for interviews. Each of these meetings will last about forty-five minutes to one hour to afford the researchers enough time to obtain the necessary information until the point of data saturation. 

#### 3.8.2. Data Collection Pre-Process

The researcher and assistant will be at the venue well on time, as O’Leary [32] believes that keeping someone waiting can be a nightmare. Rapport will be established through introductions, handshakes, small talk, and expressions of appreciation. The study will be introduced, as will its purpose and how long the interviews will take. The ethics involved will be explained, such as assurances of confidentiality, the right to decline to answer any question, and the right to withdraw from the interview upon request. 

#### 3.8.3. Data Collection Process

The research assistant will facilitate interviews by asking the right questions strategically (avoiding the pitfall of leading, offending, or confusing respondents); prompting and probing appropriately to keep the interviews moving by using phrases like ‘tell me more’, ‘really’, ‘why’, or using an inquisitive look or a few moments of silence. If audio and video recordings are found to cause uneasiness for the participants during the pre-test [32], recording of responses will be conducted through highly structured notes recorded in the spaces provided in the interview guide. Each interview per participant will end with a wind-down and closure. The wind-down will involve asking participants if there is anything else they would like to cover, contribute to, or clarify [32]. Then the interviews will end by thanking participants and asking if it is possible to contact them again if there is a need for further questions or clarification. Having the understanding that observation is a systematic method of data collection that relies on a research assistant’s ability to gather data through senses [32], in this study, not all senses will be involved in the observation process; only visual senses will be used to observe cleaning practices of cleaners at the end of the training.

#### 3.8.4. Data Monitoring 

The study will be conducted in accordance with Good Clinical Practice (GCP), as all the investigators have completed the GCP training. The Higher Degrees Committee (HDC) of the University of Venda will appoint the trial steering committee to monitor the investigation process. The principal investigator will submit quarterly reports about the progress, safety data, and endpoint of the project as prescribed in the Ethical Clearance Certificate guidelines. According to the research ethics policies of this institution, the university HDC has the authority to terminate the trial if ethical principles are violated by an investigator. The South African National Clinical Trial Registry (SANCTR) may, at its discretion, allocate an auditor on their terms for auditing trial conduct if they see fit.

### 3.9. Data Management and Analysis 

Audio-recorded data will be kept safe electronically in a password-encrypted file and will only be shared with the co-coder in the name of the research assistant. After article publication, participant data may be shared with all those whose proposals are approved by an independent review committee, provided an access agreement is signed. Papers of field notes will be kept in a locked cabinet, with restricted access to keys only to unauthorized individuals. 

Qualitative data collected from individual interviews will be analyzed using descriptive codes (to capture the characteristics of the data itself, answering who, what, where, and how the data were collected); topic/thematic codes (to describe a topic or topics from a passage); and analytical codes (explaining what the data are all about). All these analyses will be based on Tesch’s [33] open coding method, where the investigator and assistant will perform the following tasks:

- Read through all the field notes from individual interviews and observations, carefully making interpretations as they come to mind, generating a sense of the entirety of the notes, and taking notes of the ideas.
- Write interpretations as themes until all the discussions and observation data are analyzed.
- Enlist all themes, then cluster similar themes together, and arrange the themes into major, unique, and left-over themes.
- Find the most appropriate phrases for the themes, then arrange them into categories.
- Group major related themes together, drawing lines between categories to show relationships.
- Assemble the data material that belong to each category in one place [34].
- The consolidated criteria for reporting qualitative research (COREQ) [35] will be used to report the findings.

#### Strategy to Avoid Possible Subjective Interpretation Bias

Since the nature of qualitative data will make it difficult for the researcher conducting the analysis to be separated from the data, two independent coders will obtain some truth by agreement in the interpretation of participants’ responses. In addition, the participants who provided data will be asked to comment on whether the coders’ interpretations are representative of what they said or not. Peers from the Faculty of Health Sciences will also be consulted to review and comment on the conclusions if they are based on the data and codes identified.

### 3.10. Institutional Review Statement 

The study will be conducted following the Helsinki Declaration, as approved by the Human and Clinical Trials Research Ethics Committee of the University of Venda (FHS/22/PH/04/3005) in August 2023. Permission to access the selected facilities in Limpopo Province was obtained from the Limpopo Provincial Department of Health (LP_2022-05-028) in October 2023. The protocol is registered with the South African National Clinical Trial Registry (SANCTR). All changes to the protocol, such as eligibility criteria, timelines, participants, investigators, etc., will be communicated using the prescribed form to the University of Venda Research Ethics Committee. All subjects involved in the study will be requested to sign consent forms (see attached template). All data will be de-identified through code numbers and the facility alphabet for anonymity and confidentiality purposes. 

### 3.11. Results Dissemination

The intervention trial report will be compiled using the COREQ [26]. Professional language editors will be commissioned to improve the academic written language of the trial report before publication. Thereafter, the trial report will be presented at national/international conferences. In addition, the report will be published in a national/international peer-reviewed, accredited journal. Data from individual participants underlying the reported results will be made available to investigators whose proposed use of the data has been approved by an independent review committee to achieve the aims of the approved proposal, provided data requestors have signed a data access agreement. Participants and participating facilities will be given feedback on the trial through their facility/district managers.

#### Limitations 

This study sampled only one health facility per district in the province. Thus, the generalizability of the findings may be limited to participating facilities only. As soon as funding is secured, this study will serve as a pilot to inform the methodology of the bigger study, which will cover a representative sample of health facilities to cater to the generalizability of the findings to the entire Limpopo province.

## Figures and Tables

**Table 1:** Categories of health facilities in Limpopo Province, SA.

Categories of Healthcare Facilities	Total Numbers and Sample Size
Regional hospital	5—One selected—Letaba hospital representing Mopani District
Tertiary hospitals	2—One selected—Mankweng hospital representing Capricorn district
Specialized hospitals	3—One selected—Hayani hospital representing Vhembe district
PHC facilities	404—One selected Pienaarsrivier representing Waterberg district
District hospitals	31—One selected—Jane Furse representing Sekhukhune district

**Table 2:** Schedule.

Activities	March 2024	April 2024	December 2024	February 2025	March 2025
Enrolment					
Pre-test Assessment					
Intervention					
Support Visits to participants					
Intervention ends					
Post-test assessment					

## Data Availability

Audio-recorded data will be stored electronically in a password-encrypted file and will only be shared with the co-coder in the name of the research assistant and after publication with all those whose proposals are approved by an independent review committee, provided an access agreement is signed. Paper field notes will be kept in a locked cabinet, with restricted access to keys for unauthorized individuals.

## Public Involvement Statement

The reporting of participants involvement will be done against the GRIPP2 (Guidance for Reporting Involvement of Patients and the Public) checklist.

## Guidelines and Standards Statement

This manuscript was drafted against the Standard Protocol Items: Recommendations for Interventional Trials”. A complete list of reporting guidelines can be accessed via https://doi.org/10.7326/0003-4819-158-3-201302050-00583.
